# A Dynamic Transcriptional Analysis Reveals IL-6 Axis as a Prominent Mediator of Surgical Acute Response in Non-ischemic Mouse Heart

**DOI:** 10.3389/fphys.2019.01370

**Published:** 2019-10-31

**Authors:** Sally Badawi, Alexandre Paccalet, Zeina Harhous, Bruno Pillot, Lionel Augeul, Fabien Van Coppenolle, Joel Lachuer, Mazen Kurdi, Claire Crola Da Silva, Michel Ovize, Gabriel Bidaux

**Affiliations:** ^1^INSERM 1060, INRA 1397, INSA Lyon, CarMeN Laboratory, Université Claude Bernard Lyon 1, Université de Lyon, Lyon, France; ^2^IHU OPeRa, Groupement Hospitalier EST, Bron, France; ^3^Laboratory of Experimental and Clinical Pharmacology, Department of Chemistry and Biochemistry, Doctoral School of Sciences and Technology, Faculty of Sciences, Lebanese University, Beirut, Lebanon; ^4^ProfileXpert, SFR-Est, CNRS UMR-S3453, INSERM US7, University of Lyon, Lyon, France; ^5^Inserm U1052, CNRS UMR 5286, Cancer Research Center of Lyon, Lyon, France

**Keywords:** heart damage, inflammation, transcriptomics, kinetical analysis, interleukin 6

## Abstract

**Background:**

Ischemic heart diseases are a major cause of death worldwide. Different animal models, including cardiac surgery, have been developed over time. Unfortunately, the surgery models have been reported to trigger an important inflammatory response that might be an effect modifier, where involved molecular processes have not been fully elucidated yet.

**Objective:**

We sought to perform a thorough characterization of the sham effect in the myocardium and identify the interfering inflammatory reaction in order to avoid misinterpretation of the data via systems biology approaches.

**Methods and Results:**

We combined a comprehensive analytical pipeline of mRNAseq dataset and systems biology analysis to characterize the acute phase response of mouse myocardium at 0 min, 45 min, and 24 h after surgery to better characterize the molecular processes inadvertently induced in sham animals. Our analysis showed that the surgical intervention induced 1209 differentially expressed transcripts (DETs). The clustering of positively co-regulated transcript modules at 45 min fingerprinted the activation of signalization pathways, while positively co-regulated genes at 24 h identified the recruitment of neutrophils and the differentiation of macrophages. In addition, we combined the prediction of transcription factors (TF) regulating DETs with protein-protein interaction networks built from these TFs to predict the molecular network which have induced the DETs. By mean of this retro-analysis of processes upstream gene transcription, we revealed a major role of the Il-6 pathway and further confirmed a significant increase in circulating IL-6 at 45 min after surgery.

**Conclusion:**

This study suggests that a strong induction of the IL-6 axis occurs in sham animals over the first 24 h and leads to the induction of inflammation and tissues’ homeostasis processes.

## Introduction

In the widely used mouse or rat *in vivo* models of acute myocardial infarction, access to the heart requires chest opening usually following either sternotomy or rib breaking. These surgery interventions, as well as the time delay up to the end of the experiment, trigger local and systemic reaction that might act as a confounding factor when exploring the pathophysiology of ischemia-reperfusion injury. Michael et al. (1985) reported that surgical trauma induces the release of glycogen phosphorylase and creatine kinase enzymes in the lymph of dogs subjected to open-chest surgery. Nossuli et al. (2000) showed that surgical procedure induces significant variations in the expression profiles of inflammatory cytokines, such as IL-6 and TNF-α, in mouse heart. Genomic profiling of the mouse blood cells at 6 h post-surgery displayed a noteworthy change in the gene expression profiles (Coon et al., 2010). More recently, a study by Hoffmann et al. (2014) showed that sham-operated and MI animals display a similar monocyte and granulocyte circulation pattern over time, resulting in a background inflammatory response which prohibited the assessment of the MI-induced inflammatory response.

Unfortunately, myocardial transcriptomic studies seldom analyzed the gene modifications induced by the surgery procedure itself (Harpster et al., 2006; Roy et al., 2006) and to the best of our knowledge the influence of the “cytokine storm” on the modification of genes expression in cardiac cells has not been deciphered.

Therefore, we combined freely available R packages and databases in order to analyze a kinetic dataset of sham animals in order (1) to identify immune cells recruitment and differentiation in the acute phase (within first 24 h after surgery), and, (2) to identify the main cytokine/signalization pathways/transcription factors controlling the modifications in DETs. Our pipeline includes sorting out differential expressed transcripts, gene ontology analysis, time-dependent clustering of co-regulated transcripts, cross-identification of transcription factors (TFs) involved in the expression of transcript clusters and finally prediction of TFs-based protein-protein interaction (PPI) networks.

In one hand, we took advantage of the gene clustering achieved by weighted gene co-expression network analysis WGCNA (Langfelder and Horvath, 2008) to isolate the different temporal groups of transcripts prior to analyzing their GO terms and comparing them to the ones predicted from the whole list of DETs. We identified inflammation and immune responses as major biological processes that involved neutrophil, monocyte and macrophage cell markers. In the other hand, we figured out the history of DETs by retrieving the TFs most likely involved in DETs regulation prior to predicting the most probable PPI networks that could rule the activity of the highly enriched TFs. As a result, we identified a group of highly similar networks whose main characteristic was to predict a master role of interleukine-6 (IL-6) as a regulator of the selected TFs and DETs. We validated these numerical predictions by measuring IL-6 in the plasma of mice subjected to the transcriptomic analysis where a strong induction 45 min post-surgery was detected. Throughout our transcriptomic analysis and functional validation, we confirmed that surgery *per se* induces a strong inflammatory response. It also induces the recruitment of neutrophils to the myocardium and macrophages’ phenotypic changes at 24 h through the IL-6 signalization pathway.

## Materials and Methods

### Mouse Surgery Model

Male C57BL/6J mice, aged 8–12 weeks and weighing 20–30 g were obtained from Charles River Laboratories (L’arbresle, France). Mice were housed in the animal facility of the laboratory in a controlled environment with standard cycle of 12 h light/12 h dark and had free access to water and standard diet. Animals were anesthetized with pentobarbital (73 mg/kg) intra-peritoneally accompanied with (0.075 mg/kg) of buprenorphine as an analgesic. Mice were intubated orally and ventilated via a rodent ventilator. Rectal thermometer was used to monitor body temperature that was maintained within normal range by means of a heating pad. Left thoracotomy was performed and a small curved needle with an 8-0 polypropylene suture was passed, under a Euromex microscope, around the left anterior descending coronary artery. The suture was not tied and was removed after 0 or 45 min. A third group of mice underwent chest closure after 45 min and were kept alive for 24 h post-surgery.

This study was approved by the Ethics Committee of the Université Claude Bernard Lyon 1 (Approval number DR2017-48) in compliance with NIH Guide on the Use of Laboratory Animals (NIH Publication No. 85-23, revised 1996).

### Tissue Collection and RNA Extraction

Animals (*n* = 8 per time point) were randomly assigned to each group. At *t* = 0, 45 min, and 24 h post-surgery, mice were anesthetized and euthanized, hearts were harvested and the left ventricle was dissected to maximally provide the myocardium known to be at risk in ischemic hearts. Myocardium samples were placed in RNA*later* stabilizing solution (Ambion, Thermo Fisher Scientific) and stored at −80°C until use.

RNA was extracted by Tripure reagent solution (Roche), treated with Proteinase K (Qiagen) and purified by RNeasy Mini kit (Qiagen) where DNase I (Qiagen) is treated on column. RNA purity, quantity and integrity were assessed both by spectrophotometry (NanoDrop ND-1000, NanoDrop Technologies) and nanoelectrophoresis (2100 Bioanalyzer, Agilent Technologies). RNA purity: A_260__/__280_ ∼ 1,8 and A_260__/__230_ ∼ 2 and RNA integrity number: 8–10.

### RNA Sequencing

Purified RNA samples were provided to ProfileXpert, Inc., for library construction and sequencing. Quality and quantity checks were performed by means of Fragment Analyzer (Agilent) and QuantiFluor RNA dye (Promega). Library construction was carried out using NextFlex Rapid Directional mRNA-Seq (Bioo-Scientific, PerkinElmer Company) following the manufacturer protocol. Libraries were applied to an Illumina flow cell High and run on the Illumina Nextseq 500 as a single end read for 76 pb. On average, 12 samples were loaded to each flow cell. Image analysis and base calling was carried out using the NCS 2.0.2 and RTA 2.4.11 Illumina software suite implemented on the Illumina sequencing machine. Final file formatting, demultiplexing, and fastq generation were carried out using Bcl2fastq v2.17.1.14.

### Bioinformatic Analysis

Trimming of reads was performed using cutadapt v1.9.1 software (Martin, 2011). Then the reads were mapped to the mm10 genome using TopHat v2.1.0 (Kim et al., 2013) software with default parameters (bowtie 2.2.9; Langmead and Salzberg, 2012). Reads were counted using htseq-count v0.6.0 software to generate raw counts.

Several pipelines and software packages have been developed to aid in the management and analysis of the high throughput data. These packages differ considerably in their analytical pipeline, statistical model (Robinson and Smyth, 2007; Tarazona et al., 2015) and normalization tool (Bullard et al., 2010; Robinson et al., 2010; Trapnell et al., 2012; Rapaport et al., 2013; Love et al., 2014; Evans et al., 2017). The choice between the normalization methods strongly influences the differential expression analysis (Burden et al., 2014), where EdgeR and DESeq2 are the most frequently used ones (Lamarre et al., 2018). In our study, differentially expressed transcripts (DET) were computed with DESeq2 (Love et al., 2014) package version 1.20.0 supplied by R software (version 3.4.4) (R Core Team, 2019) via a likelihood ratio test implemented in *DESeq* function. For all the analysis, we kept transcripts with FDR less than 0.05 corrected via the Benjamini–Hochberg method (Benjamini and Hochberg, 1995).

Transformed counts by DESeq2 were visualized with a principal component analysis (PCA) [prcomp()] in R. PCA is a dimensionality reduction method that maximizes the variability explained by the newly formed dimensions. 1–3 dimensions known by principal components (PC) can be chosen to represent the data, where each PC is orthogonal to the other (Abdi and Williams, 2010; Lever et al., 2017).

To construct transcript co-expression network, we ran the *cutreeDynamic* function of the (WGCNA) R package (version 1.63) (Langfelder and Horvath, 2008) on the matrix of normalized counts for the DETs identified with DESeq2.

The matrix of normalized counts was computed with the *varianceStabilizingTransformation* function of the DESeq2 package. WGCNA applies PCA where the first PCs of each formed module are called eigengenes. Soft threshold (beta) that represents the exponential parameter for power law distribution was chosen based on a scale free topology criterion. Adjacency matrix was constructed and then transformed into a topological overlap matrix. Transcripts were hierarchically clustered using the *flashClust* function and clusters of transcripts having similar profiles, referred to as modules, were formed.

### Gene Ontology Analysis

Differentially expressed transcripts were subjected to functional enrichment analysis by STRING software version 10.5^1^ (Franceschini et al., 2013). Hypergeometric tests are used to identify enriched terms that are sorted by their FDR. GO terms with FDR < 10^–4^ were kept for further analysis. We have proposed a score, named *z*-score (*z*), to measure the enrichment of the GO terms in modules as follows:

(1) $$z=\frac{\left(x-0.5-\frac{B\times n}{N}\right)}{\sqrt[{2}]{n\times \frac{B}{N}\times }\,\left(1-\left(\frac{B}{N}\right)\right)}$$

x: count of observed transcripts in each GO term of module.

B: count of observed transcripts in each GO term of DETs.

*n*: total count of observed transcripts in module.

N: count of DETs.

Our *z*-score estimates whether a given GO term is enriched in a gene module compared to all DETs and is thus assessing whether a module is clustering genes involved in a common biological process. We assumed that a GO term is enriched in a certain module if *z*-score > 2.

### Transcription Factor Enrichment Analysis

oPOSSUM 3.0 (Kwon et al., 2012), a freely available web accessible software was used to identify enriched transcription factors binding sites (TFBSs) in the 5000 bp upstream and downstream sequence of the DETs. Default search parameters of the single site analysis were kept as they are originally set in the software. oPOSSUM performs an exact Fisher test which measures the probability of a non-random association between the co-expressed gene/transcript set and the TFBS of interest and calculate a *F*-score as equal to −ln(*p*-value). *F*-score is thus assessing the probability that at least one TFBS would be significantly associated with the observed transcript list. Besides, a *z*-score is calculated using the normal approximation to the binomial distribution to compare the rate of occurrence of a TFBS in the target set of genes/transcripts to the expected rate estimated from the pre-computed background set. Thus, the *z*-score is estimating the specific enrichment of given TFBS in the gene/transcript set compared to background gene set. TFs were ranked by *F*-score and *z*-score, respectively and were clustered into five groups.

### Functional Network Inference

Protein-protein interaction (PPI) networks were generated using the freely available STRING database version 10.5 (Franceschini et al., 2013). Clustered TFs were inputted separately in STRING to generate background networks based on the predicted associations between these TFs (curated or experimentally determined interactions). Resulting TF-PPI networks were expanded into several layers (shells) via sequentially adding predicted associated proteins (nodes) until networks of five layers were generated as explained in Supplementary Figure S6. During each step, GO terms with FDR < 10^–4^ were assigned to major biological processes. The major biological processes were then normalized to the total number of identified GO terms in each network. To find out how similar is the processes’ prediction to that of DETs, shared biological processes between TF-PPI network and DET were sorted out and then were normalized by the total number of shared GO terms. TF-PPI networks that recapitulated the greatest number of biological processes obtained from the DETs were selected as being the best putative TFs-regulated networks.

### Isolation of Cardiac Resident Cells

Zero minutes and 24 h post-surgery, mice (4 per time point) were injected intra-peritoneally with 50 UI/kg heparin sodium for 10 min. Mice were euthanized and heart was harvested and cannulated through the aorta. Afterward, a small clip was attached to the aorta’s end and a thread beneath it was tied to prevent the heart from falling (O’Connell et al., 2007). The heart was firstly perfused for 3 min with the perfusion buffer [NaCl 120.4 mM, KCl 14.7 mM, KH_2_PO_4_ 0.6 mM, Na_2_HPO_4_ 0.6 mM, MgSO_4_-7H_2_O 1.5 mL, Na-HEPES 10 mM, NaHCO_3_ 4.6 mM, Taurine 30 mM, 2,3-butanedione monoxime (BDM) 10 mM, and Glucose 5.5 mM, pH 7.0]. The latter was replaced by digestion buffer (50 mL of perfusion buffer and Collagenase II 2.4 mg/mL) for 2 min 30 s. 100 mM CaCl_2_ (final concentration 40 μM) were then added. Perfusion was continued for 6 min and 30 s. During the entire procedure, the heart was perfused at 4 mL/min rate and the solutions were maintained at 37°C to mimic physiological conditions. After 12 min, the heart was removed and placed in a 100-mm dish. It was then cut into small pieces that were placed in a tube containing myocyte digestion buffer. Small pieces were gently pipetted several times to ensure the complete myocardium digestion. Digestion stop buffer [45 mL of perfusion buffer, 5 mL of fetal bovine serum (10%), and 6.25 μM of 100 mM CaCl_2_ (12.5 μM)] was then added up to a final volume of 10 mL. The tube was then centrifuged for 3 min at 20 *g* at room temperature (Eppendorf 5810R). Supernatant was collected and centrifuged for 5 min at 500 *g*. Cells pellet was used for labeling protocol.

### Flow Cytometry Analysis

#### Labeling Protocol

Cell pellets were re-suspended in phosphate buffer saline (PBS) and divided in two tubes: Labeling and isotypic control. PBS was added in each tube and tubes were centrifuged 5 min at 500 *g* and the supernatant was discarded. FCR blocking solution (diluted FCR: 1:10 in PBS; 100 μl/tube of dilute FCR solution; FCR blocking mouse reagent, Miltenyi biotec, 130-092-575) was then added for 10 min at 4°C. Following incubation, PBS 0.5% BSA (Bovine Serum Albumin) was added and tubes were centrifuged 5 min at 500 *g*. The two tubes were incubated for 30 min at 4°C in dark with the isotopic or the labeling solution. Incubation was stopped with the addition of PBS 5% BSA followed by a 5 min centrifugation at 500 *g*. Supernatants were discarded and pellets re suspended in PBS before flow cytometry analysis.

#### Samples’ Processing and Data Analysis

Flow cytometry experiments were conducted using Fortessa X-20 equipped with four lasers and 16 fluorescent detectors. Markers of macrophages (CD11B, F4/80, CD206, and CD86) and neutrophils (Ly6g) were analyzed after immunostaining (antibodies are listed in Supplementary Table S1) in order to estimate the proportion of each population. 100,000 of total events were acquired for each condition. Data were analyzed by DIVA Software (BD Biosciences). The percentage of each cell subtype was calculated after the multiple gating of the different fluorescent markers. The sorting of macrophage subtypes was performed as followed: CD11b+ and/or F4/80+ positive cells were gated and represented the total macrophage population. Within this cell population, the percentages of type 1 macrophages (M1) (CD206−/CD86+), type 2 macrophages (M2) (CD206+/CD86−), M1 + M2 (CD206+/CD86+) and negative M1 + M2 (CD206−/CD86−) macrophages population were figured out. Percentage of M1, M2, and double M1 + M2 phenotypes were normalized by the sum of these three population to assess the shift in differentiation.

### Plasma Preparation and IL-6 Assay by ELISA

After anesthesia and prior to euthanasia, blood samples were collected from the inferior vena cava. Blood was centrifuged at 500 *g* for 5 min at room temperature to isolate plasma. The latter samples were stored at −80°C for later use. Interleukin 6 (IL-6) concentrations were measured in plasma samples by the enzyme like immunofluorescent assay (ELISA) using the Mice IL-6 ELISA Kit (R&D Systems, Minneapolis, MN, United States) based on the manufacturer instructions. The sensitivity test was 1.8 pg/mL.

### Statistics

In order to determine the number of mice per group, a power analysis was performed with G^∗^Power (version 3.1.9.2) (Faul et al., 2007) with the following conditions: One-way ANOVA parameters with alpha = 0.05, beta = 0.2, and effect size = 0.7 with three groups (0 min, 45 min, 24 h).

Statistical analysis were performed with One-way Anova (Tukey’s multicomparison test and Kruskal–Wallis non-parametric test), student *t*-test, two-way anova tests and spearman correlation analysis were performed with Graphpad Prism (version 7.0a) (GraphPad Software, La Jolla, CA, United States)^2^.

### Data Availability

All sequencing datasets used in this study are submitted in international public repository, Gene Expression Omnibus, under accession identification as GSE127244.

## Results and Discussion

### Experimental Design

A methodical issue to assess the sham effect relies on the complexity of the analysis of OMICs dataset. These latter are very sensitive to the statistical power of the study, the experimental design and the accuracy of the analytical pipeline of OMIC studies. The experimental design of dynamic transcriptomic studies is largely affected by factors as the precision of measures, ethical rules, expenses’ constraints and statistical power. The precision of measures is, however, highly dependent on the biological variability of the studied system and the experimental error introduced by the surgery effect or the RNA-seq protocol (Conesa et al., 2016). Another type of errors originates from static comparisons between “pretreatment” and “post-treatment” samples which can neither correctly recapitulate the dynamic of a pathology nor enable the characterization of the molecular cascade ruling the modification of gene/transcripts expression (Kim et al., 2012; Chang et al., 2015). Unlike static analysis, kinetical analysis permits the identification and clustering of gene/transcripts based on their expression profile. This list of DETs must be thought as temporal signature which can be used to predict the future evolution of the cellular systems or to infer the molecular mechanisms which controlled and induced these modifications in DETs expression level.

We profiled the associated effects of anesthesia, thoracotomy, removal of the epicardium and suture passed under the left anterior descending artery on the change of gene expression profiles in mouse myocardium. Dynamics of transcripts expression were quantified by mRNA sequencing at three different time points post-surgery (0 min, 45 min, and 24 h). This procedure is similar to the “Sham” procedure used in mouse model of myocardial infarction explained by Harisseh et al. (2017). Unlike blood, temporal analysis in organs requires sacrificing different animals to cover all time points. Consequently, the variance of gene/transcripts expression over time is mixed with the inter-individual variance. Therefore, the sensitivity of the analytical pipeline to detect DETs is highly affected by the statistical power of the study. The experimental design considered a total of 24 animals (eight mice per time point) to meet the statistical parameters which are presented in the section “Materials and Methods.” Surprisingly, in several recent dynamic transcriptomic studies that are involved in ischemia reperfusion (Roy et al., 2006; Kim et al., 2012, 2018; Prat-Vidal et al., 2013; Andreeva et al., 2014; Khan et al., 2017), no power test was carried out to optimize the number of samples, which may have led to the underestimation of the number of DETs. Analytical comparison studies have estimated that a minimum of five and six replicates per condition is required to obtain stable significant results in microarray and RNA sequencing experiments, respectively (Pavlidis et al., 2003; Schurch et al., 2016; Lamarre et al., 2018).

Considering the studies which compared analytic tools for transcriptomic (Rapaport et al., 2013; Soneson and Delorenzi, 2013; Burden et al., 2014; Seyednasrollah et al., 2015) and seeking a method with the highest sensitivity for controlling the false discovery rate (FDR) (Love et al., 2014) and compatible for the analysis of kinetics, we have decided to work with DESeq2 analysis method. The analytical pipeline followed in this study is presented in Supplementary Figure S1.

### RNA-Seq Data Processing and Analysis

**FIGURE 1:**
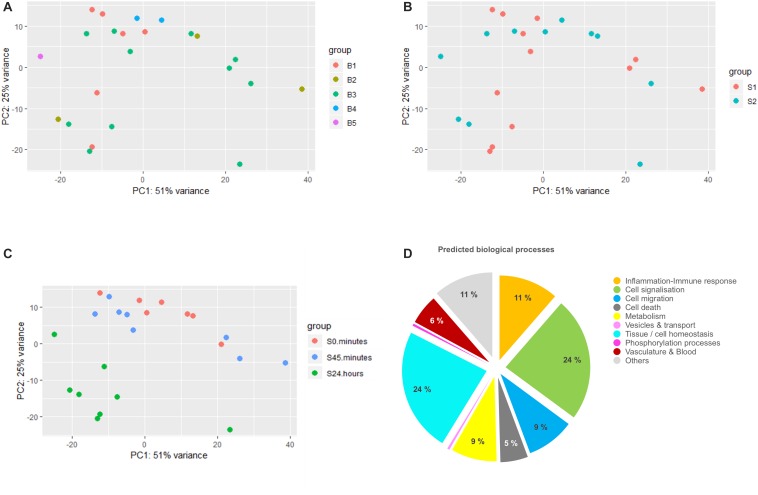
Differential analysis and gene ontology analysis. **(A–C)** represent principal component analysis (PCA) plots of the data clustered by surgeon (S1 and S2), batch (B1–B5), and time (0 min, 45 min, and 24 h), respectively. Batch corresponds to the different pools of library construction during sequencing. **(D)** Pie chart showing the major biological processes expressed as the percentage of GO terms detected in the gene ontology analysis of the differentially expressed transcripts list.

DESeq2 uses a generalized linear model (GLM) with a Negative Binomial distribution to model the counts associated with a given gene. Compared to the classical Poisson count distribution the Negative Binomial distribution can account for over dispersion in the data (variance higher than the mean). To be able to estimate both parameters of the distribution for each gene, the variance distribution is computed from a mean variance function fitted across all genes. DESeq2 takes raw reads as input but uses a sequencing depth offset parameter (Love et al., 2014). Firstly, we assessed the different possible sources of experimental error: different surgeons and different cDNA libraries in the mRNA seq process (defined as “Batch effect”). Data visualization by PCA in Figures 1A–C clearly shows that our samples were clustered neither by the surgeons nor by the batch. Samples were actually clustered by their variation over time, where “24 h post-surgery” condition was responsible for the highest variation in the data. 0 and 45 min samples were grouped together indicating no major effect of the surgery after 45 min in comparison to 24 h. Noteworthy, the three groups were scattered along PC1 and PC2, what suggested at least two different sources of variance. In addition, PCA plot did not show any outlier samples that might affect the analysis, noting that samples are spread out along PC1 and display a large within-group variability that might be of biological and technical origin we cannot control. Among the 27,661 non-zero transcripts, DESeq2 yielded 1209 DETs over time with FDR <0.05 (List of transcripts is available in Supplementary Table S2).

To identify the major biological processes affected in response to surgical intervention, functional enrichment analysis was performed using STRING software. We first proceeded with the GO analysis of the complete DET list that had generated a list of more than 700 significantly enriched GO terms. To reduce their dimensionality, we arbitrarily selected a cut-off for the FDR of GO terms below 10^–4^ and then kept 228 GO terms for the rest of the analysis. We next classified them into bigger biological processes in order to understand what were the consequences of the surgery. As summarized in Figure 1D, we identified the major biological processes as: cell signalization (24% of GO terms), tissue regeneration (including mechanisms of cell homeostasis, tissue organization, embryogenesis-related processes, wound healing and representing 24% of GO terms), inflammatory and immune response (11% of GO terms), cell migration (9% of GO terms), metabolism (9% of GO terms), cell death (5% of GO terms) and processes involved in vasculature remodeling (6% of GO terms).

In summary, the 1209 identified DETs were associated with biological processes among which some were expected like inflammation and immune response. Although, this coarse-grained strategy is broadly used to investigate gene network response to stimuli, it is highly dependent on the quality of GO annotation, the rationality of GO selection and clearly lacks understanding of both the cell network (including gene and protein) organization and its modification over time. We therefore aimed to temporally and phenotypically organize the DETs in order to isolate different regulation waves of gene network.

### Weighted Gene Co-expression Network Analysis

**FIGURE 2:**
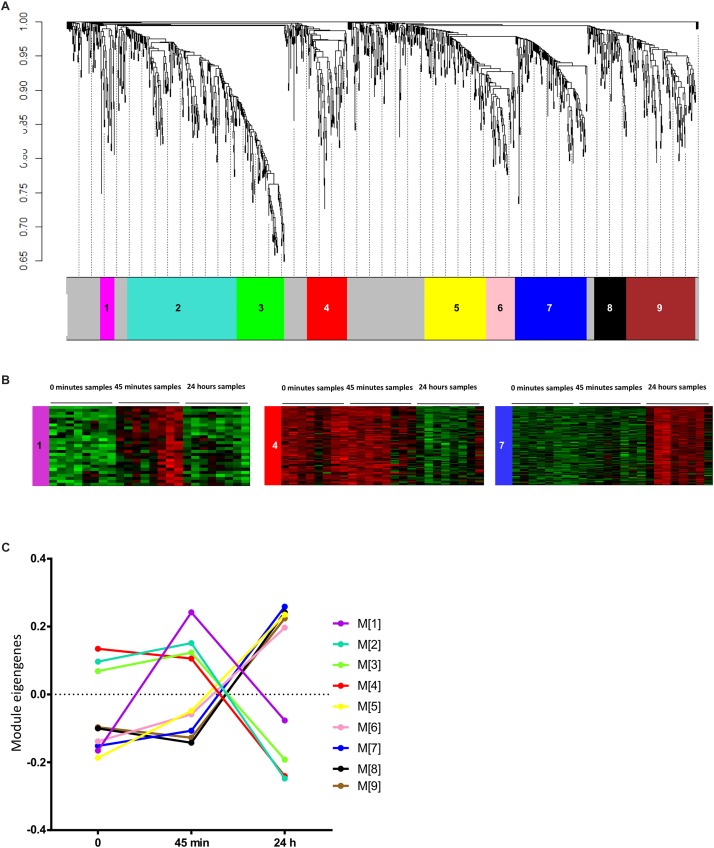
Weighted gene co-expression network analysis. **(A)** Gene dendrogram representing the hierarchical clustering of transcripts based on their similarity in the expression profiles. Tree branches correspond to transcripts and colors underneath the tree corresponds to the modules assignment by Dynamic Tree Cut of the WGCNA package. **(B)** Heatmaps of the level of expression of transcripts assigned to examples of the three main time profile of transcripts expression: the magenta (M[1]), red (M[4]), and blue (M[7]) modules. Red color corresponds to higher expression and green color corresponds to lower expression. **(C)** Line graph representing the variation of the expression profiles in the different modules over time. Values represent the mean eigengenes figured out by WGCNA. The color code used is the same as in **(A,B)**.

First, we looked for co-varying transcript signatures and aimed to predict their associated biological processes. Weighted gene co-expression network analysis (WGCNA) is an unsupervised analysis that aims to construct modules (clusters) of highly correlated transcripts according to the similarity in their expression profiles (Langfelder and Horvath, 2008). WGCNA was performed on the1209 DETs. Prior to analysis, we checked for outliers’ samples to exclude, but none was detected as displayed in Supplementary Figure S2A. The soft threshold was set to 30 based on the scale free topology criterion (Supplementary Figures S2B,C). Transcripts were hierarchically clustered (Figure 2A) and nine clusters of transcripts with similar profiles were formed. List of transcripts assigned to each module is available in Supplementary Table S3. For a better understanding, a color code was assigned for each module of transcripts. The size of these modules ranged from 27 to 210 transcripts and 300 (24.8% of DETs) were not assigned to any module, colored in gray. As examples of the different profiles obtained in these modules, heatmaps representing the expression profiles of the transcripts assigned to the magenta (M[1]), red (M[4]) and blue (M[7]) modules were displayed in Figure 2B, where remaining modules’ expression profiles are displayed in Supplementary Figure S2D. WGCNA summarizes the distribution of transcripts expression via PCA and the first PC is called “eigengene.” Eigengene values of transcripts modules were calculated for each sample and eigengene means were plotted for each module over time in order to summarize the average variation in transcript expression (Figure 2C). Interestingly, we identified three major time profiles: the magenta module (M[1]) was clustering transcripts transiently induced at 45 min, modules 2–4 clustering transcripts with a decrease at 24 h and modules 5–9 with an increase at 24 h. We hypothesized that, since modules have been clustered with positively co-varying transcripts, they could unlikely predict non-linear biological processes spanning a broader range of time. We thus tested whether the different combinations of the three modules with the greater number of GO terms: [M1], [M6], and [M7] could enhance the predictions.

**FIGURE 3:**
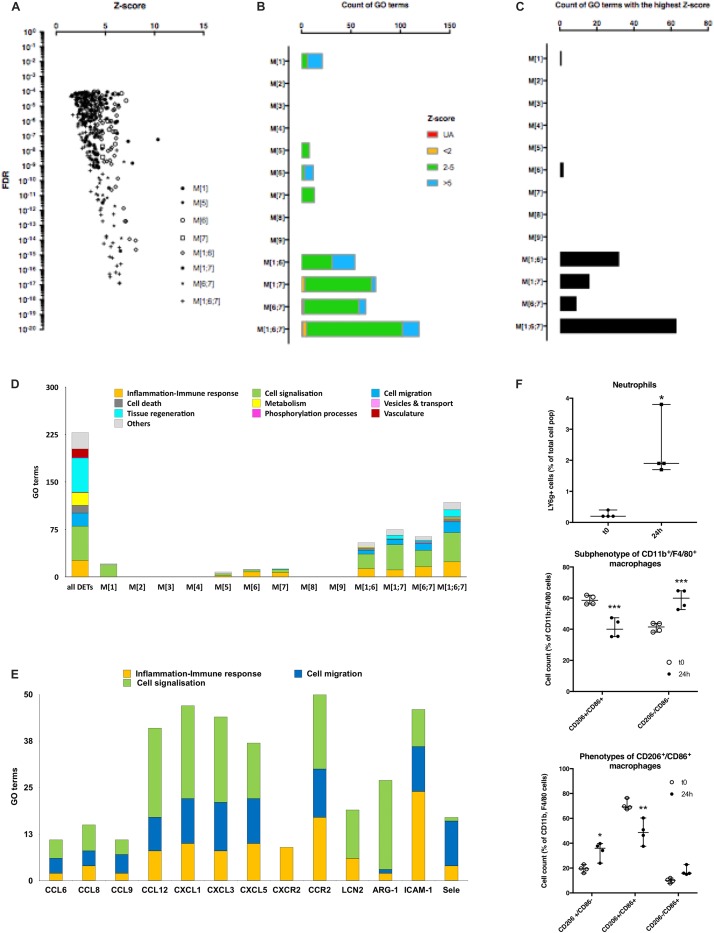
Analysis of GO terms enrichment and characterization of immune cells. **(A)** Scatter plot of *z*-score and FDR of GO terms predicted from different transcripts modules (reported as [module number]) and combination of modules (reported as [module number; module number]). Values were filtered for FDR < 10^– 4^ and enrichment factor (*z*-score) > 2. **(B)** Bar graphs displaying the count of the filtered GO terms for different groups of transcripts modules and which are shared in the list of GO terms from DETs. Blue and green colors represent the enrichment score of the terms present in the DET’s GO terms list and the red color represents the unassigned terms (UA). **(C)** Bar graph showing which transcripts’ modules recapitulate the best GO terms observed in DETs. Each GO terms accounting in **(B)** were counted only once in the module showing the highest *z*-score value for this GO term. **(D)** Histogram plot representing the count of GO terms classified into bigger processes displayed by colors for all DETs and the different groups of modules. **(E)** Histogram plot representing the count of inflammation/immune response, cell signalization, and cell migration processes’ GO terms constituting some inflammatory transcripts of M[1;6;7] group of modules displayed by colors. **(F)** Dot plots showing the percentage of different cell populations: LY6g + neutrophils (top panel), CD206-/CD86-macrophages (middle panel) and M1, M2, and M1 + M2 macrophages (lower panel) at 0 min and 24 h post-surgery (^∗^*P* ≤ 0.05, ^∗∗^*P* < 0.01, and ^∗∗∗^*P* < 0.001).

In order to predict the biological processes related to these modules and assess the strength of this prediction, we first studied the distribution of transcript counts as a function of GO term counts for all groups of transcripts modules and for all DETs as well (Supplementary Figure S3). Expectedly, the high transcripts counts in the DETs were correlated with the greatest number of GO terms. However, a strong discrepancy was observed for the groups of modules. For instance, M[2] including 210 transcripts (Supplementary Figure S3) had no significant predicted GO terms whereas group M[1;6] including 83 transcripts had 240 predicted GO terms and group. This firstly suggested that a greater number of transcripts did not mandatorily mean a great count of GO terms and thus no size artifact occurred in the comparison between modules and combinations of modules. We next quantified to which extent modules or combinations of modules may be strong predictors of the DETs-based GO terms. This feature, defined as GO term enrichment (see the section “Materials and Methods”), reflects the fact that modules might sort out transcripts related to similar biological processes. All GO terms of all modules or combination of modules were first filtered with FDR < 10^–4^ and then selected if their enrichment factor was >2 (Figure 3A). List of enriched GO terms and their FDR for the different modules and combinations are available in Supplementary Table S4. The count of the selected GO terms for each modules or combination of modules is presented in Figure 3B. Next, we sorted out the groups of modules having the highest enrichment factor for each and every GO term (Figure 3C) in order to estimate which module or combination of modules achieved the strongest predictions of GO terms. Six groups of modules were sorted out and were considered as the best predictors based on their shared GO terms with the DET-derived ones, where among them group M[1;6;7] had the highest count of the highest-enriched GO terms.

Filtered GO terms were classified into bigger biological processes (Figure 3D). M[1], which was transiently induced at 45 min (Figure 2C), was mainly involved in cell signalization process what we interpreted as a transcriptional response following the surgery to maintain cells’ homeostasis via the shift in the state of different signalization pathways. Combinations of modules M[1;6], and M[6;7] looked similar and predicted the induction of signalization pathways, cell migration and inflammatory/immune response, while combinations M[1;7], and M[1;6;7] predicted the induction of signalization pathways, cell migration, tissue regeneration and inflammatory/immune response (Figure 3D). As anticipated from the calculation of the highest-enriched GO terms, the combination of modules M[1;6;7] was the one recapitulating the highest predictions from the DETs: signalization pathways (46 vs. 54 GO terms, respectively), cell migration (17 vs. 21 GO terms, respectively), cell death (5 vs. 12 GO terms, respectively) and inflammatory/immune response (24 vs. 26 GO terms, respectively). However, tissue regeneration was poorly recapitulated (11 vs. 54 GO terms, respectively) while neither metabolism nor vasculature were detected. This suggested that this method was efficient for classified and enriched transcripts associated with some of the DETs features but not all.

Eventually, looking at the transcripts of M[1;6;7], we found many inflammatory markers including chemokines (CCL6, CCL9, CCL12, CXCL1, CXCL3), chemokine receptors (CXCR2 and CCR2), adhesion markers of endothelial cells (ICAM-1 and Sele) in addition to markers of neutrophils and macrophages (LCN2 and ARG-1, respectively) (Frangogiannis, 2002; Sadik et al., 2011; De Filippo et al., 2013; Arango Duque and Descoteaux, 2014). We then checked the occurrence of each of these transcripts in the GO terms associated with inflammation/immune response, cell signalization and cell migration biological processes of M[1;6;7], we found that most of them were actively involved in these three main processes (Figure 3E). In this regard, we hypothesized that transcripts related to inflammatory/immune response, cell migration and transcripts involved in cell signalization could have fingerprinted the recruitment and/or the differentiation of immune cells within the myocardium.

We assessed the quantification of macrophages and neutrophils in mouse heart subjected to surgery by FACS analysis. A significant 9.3-fold increase of the percentage of high LY6g-positive cells was found in non-myocytes cell extract from the myocardium 24 h post-surgery, this suggested an increase in the population of neutrophils (Figure 3F and Supplementary Figure S4A). We observed a shift in the phenotypes of F4/80^+^ and CD11b^+^ macrophages 24 h post-surgery. First, an increase in CD206^–^/CD86^–^ cells was observed (from 41.2 ± 2.9 to 59.3 ± 6.3% of F4/80^+^/CD11b^+^ macrophages; adjusted *p*-value < 0.0001) as reported in Figure 3F. This suggested the recruitment of monocytes to the myocardium. Second, excluding the double negative population of macrophages (CD206^–^/CD86^–^), a transition of double CD206^+^/CD86^+^ (from 70.6 ± 4.0 to 48.8 ± 9.5%; adjusted *p*-value: 0.0013) to either type M1 (from 10.0 ± 1.8 to 17.4 ± 3.6%; adjusted *p*-value: 0.2778) or type M2 (from 19.4 ± 2.9 to 33.9 ± 7.0%; adjusted *p*-value: 0.0181) was detected (Figure 3F and Supplementary Figure S4B). This double CD206^+^/CD86^+^ phenotype was previously reported in the heart (Walter et al., 2018); however, it was not shown whether these cells could evolve to single CD206+ or CD86+ phenotype over time.

Altogether, our results confirmed the recruitment of neutrophils and F4/80^+^/CD11b^+^/CD206^–^/CD86^–^ macrophages/monocytes and showed a possible differentiation of CD206^+^/CD86^+^ macrophages into type2 macrophages (M2) to the mouse heart within the first 24 h post-surgery. Tissue regeneration, metabolism, phosphorylation processes which were major components of the DETs-based GO terms were only found, and at low percentage, in the groups M[6;7] and M[1;6;7] (Figure 3D). We thus looked for a complementary strategy in order to predict the biological pathways involved in the regulation of these biological processes.

### Transcription Factor Enrichment Analysis and Gene Network Inference

**FIGURE 4:**
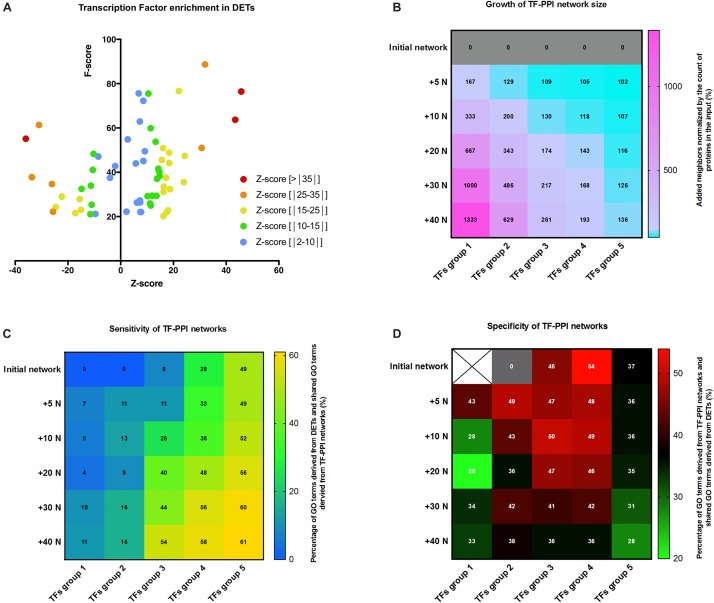
Transcription factor analysis and PPI networks simulation. **(A)** Scatter plot of *z*-score and *F*-score of the over-represented transcription factor binding site (TFBS) detected in genes of the DET list and generated by oPOSSUM. Only TFs with *F*-score above 20 are considered. TFs were classified by their *z*-score as depicted in the legend. **(B)** Heat map showing the growth of TF-PPI network size as calculated by the percentage of increase in the number of proteins at each step of network growth: +5, +10, +20, +30, and +40 neighbors (+5 N; +10 N; +20 N; +30 N; +40 N). TF-PPI networks were simulated from each different TFs groups as input. TFs group were: group 1 (*z*-score > | 35|), group 2 (*z*-score > | 25|), group 3 (*z*-score > | 15|), group 4 (*z*-score > | 10|), and group 5 (*z*-score > | 2|). **(C)** Heat map displaying the sensitivity of the networks based on the growth (increase in number of neighbors) and the input (groups of TFs) of TF-PPI. Color gradient displays the percentage of GO terms of all DETs shared with GO terms derived from TF-PPI network and values indicates the percentage. **(D)** Correlation matrix between the growth (increase in number of neighbors) and the input (groups of TFs) of TF-PPI networks reporting the specificity of the networks. Color gradient displays the percentage of GO terms of TF-PPI network shared with the DET-based GO terms and values indicates the percentage.

Transcriptomic signature could be considered as the outcome of a response to a stimulus carried by molecular pathways. Retro-analysis could thus be used to delve into the molecular history of the biological system. We started assessing which transcription factors could have likely regulated DETs and transcripts’ modules. Over-represented transcription factor binding site (TFBS) in the promoter sets of the DETs using oPUSSUM software was calculated by the mean of *z*-scores and fisher exact test scores. *Z*-score measures the change in the TF motifs of the target set compared to the background set, whereas the fisher score assesses whether the genes associated with the TF is greater than what would be expected by chance (Kwon et al., 2012). We first found that transcripts modules were mainly able to predict the TF outliers predicted from all DETs like Klf4, SP1, STAT3, and NFKB1 (Supplementary Figure S5). Noteworthy, Klf4 and SP1 are two transcription factors which have been reported to play an inflammatory role, more specifically a role in macrophage activation and polarization (Feinberg et al., 2005; Lee et al., 2005; Liao et al., 2011; Karpurapu et al., 2014). This could mean that these two transcription factors reported mainly modifications in cell subtype rather than changes in the genotype of resident cells. Besides, we noticed that both *F*-score and *z*-score values of TF enrichment dropped to low values for transcripts’ modules as compared to those predicted from all DETs (Supplementary Figure S5). In order to keep the strongest TFs prediction possible, we thus chose to work only with TF enrichment from all DETs. Since no objective threshold can be applied to *z*-score and *F*-score, transcription factors were filtered for *F*-score below 20 (*p*-value = 2.06 × 10^–9^) and thus sorted out by *z*-score in five groups cumulating more and more TFs with lower *z*-score values (Figure 4A). The next step of our retro-analysis considered that TFs activity was driven by a response to stimuli via signalization pathways. We thus took advantage of the protein-protein interaction (PPI) networks simulated with STRING which used the different TFs groups as inputs. The first TF-PPI network was defined as the initial network and it only relied on the interplay between the TFs.

In the second step, STRING added a first layer of either 5 (+5N) or 10 (+10N) neighboring proteins being the most likely interactors of the TFs and thus it built the first layer PPI network (+5N), named first layer of growth. This operation was repeated consecutively five times. An example of the initial, first layer and second layer PPI networks performed for TFs group 3 are displayed in Supplementary Figures S6A–C. TF-PPI networks were built on an increasing number of proteins from the initial layer to the fifth layer (+40N) (Supplementary Figure S7) which, however, depending on the count of initial proteins represented different growth of the network (Figure 4B). We considered that a useful network added enough information (neighbors) to predict the mechanism upstream TFs (receptor, signalization pathways). In the third step, GO terms predicted for each TF-PPI network (simulated GO) were retrieved, filtered (FDR < 10^–4^) and compared with GO terms of DETs (experimentally derived GO) to determine the shared GO terms between simulated data and experimental data. First, we figured out the sensitivity of the TF-PPI network built by measuring the proportion of all DETs-predicted GO terms shared in the different TF-PPI networks. The correlation matrix reported that TF-PPI network set with a too great input (TFs group 5) saturated between 49 and 61% of all DETs-predicted GO terms regardless of the addition of neighbor proteins (Figure 4C). This made this kind of TF-PPI network unable to predict mechanisms upstream of TFs activation. Conversely, TF-PPI networks set with a too low input (TFs group 1 and 2) only found 16% all DETs-predicted GO terms and were thus unable to simulate a network that could be thought to predict the mechanisms leading to DETs. However, TFs groups 3 and 4 were able to retrieve between 8 and 58% of all DETs-predicted GO terms. This highlighted that a correct balance between input and growth of TF-PPI network was required to gradually find simulated GO terms in DETs-derived ones. We next figured out the specificity of TF-PPI-networks by calculating the proportion of GO terms predicted in the TF-PPI network which were shared with all DETs-predicted GO terms. The correlation matrix shown in Figure 4D demonstrated that the growth of TF-PPI networks decreased their specificity. An optimal specificity was reached at the first growth for TFs group 1 and 2, was almost stable from initial network to the third growth of the networks for TFs group 3 and 4 and was low for any growth of the TFs group 5. Altogether, these results demonstrated that the highest specific TF-PPI networks were TFs group 3 and 4 with growth from the initial to the third layer (Figure 4D). The most dynamic sensitive networks having percentage of shared GO terms above 40% were found for TFs group 3 and 4 with growth from the third to the fifth layer (Figure 4C) and that the network growth was above 150% for conditions below a diagonal starting from first layer of growth TFs group first to fourth layer of growth TFs group 4 (Figure 4B). From these parameters, we found that third layer of growth TFs group 3 shown the balance between specificity, sensitivity and network growth and was selected for the further analysis.

**FIGURE 5:**
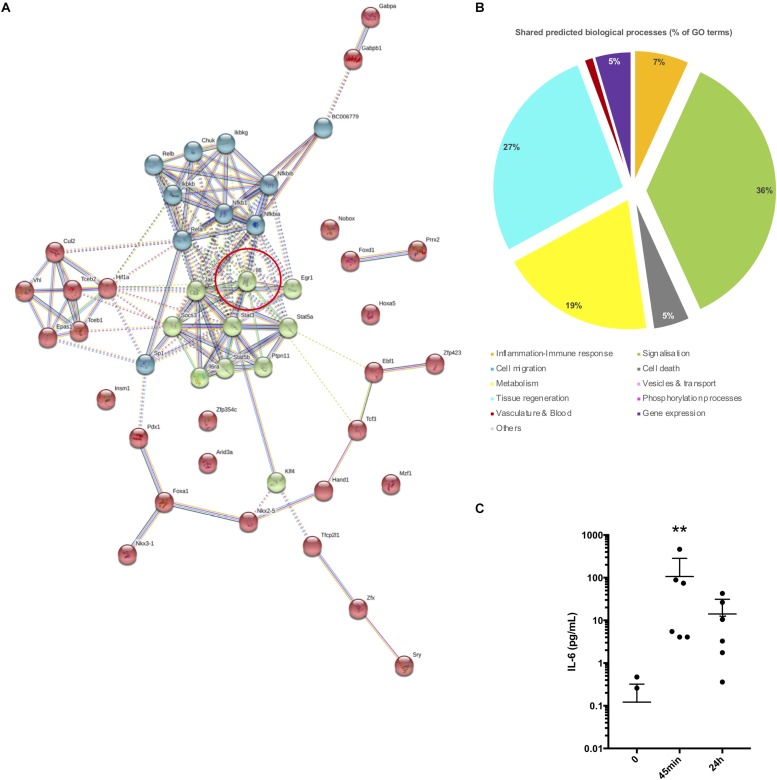
Transcription factor analysis and PPI networks simulation. **(A)** TF-PPI network simulated by STRING from TFs group 3 and expanded for three layers (+20 neighbors). Line connections between proteins displaying the type of interaction. Red circle reports the central position of interleukin 6 in the network. **(B)** Pie chart showing the major biological processes expressed as the percentage of GO terms. GO terms predicted from the TF-PPI network shown in **(A)** and shared in the list of GO terms derived from were taken for this analysis. **(C)** Plot representing IL-6 concentration in the plasma of mice at 0 min, 45 min, and 24 h post-surgery (*n* = 6 per time point) (^∗∗^*P* ≤ 0.05).

A branched network representation of third layer of growth TFs group 3 PPI network is displayed in Figure 5A. K-means clustering was used to highlight three different portions in this network (Figure 5A): core network in green which shares maximum connections with the other part of the network, secondary network in blue and peripheral network in red. GO terms associated to this network were filtered (FDR < 10^–4^) and then clustered in biological process expressed as percentage of the total of GO terms (Figure 5B). Cell signalization (36%), tissue regeneration (27%), metabolism (19%), inflammatory/immune response (7%), and cell death (5%), and vasculature (2%) were the main processes supported by the shared GO terms. Interestingly, these functions could support wound healing and tissue homeostasis in response to stress and thus could involve the cardiac resident cells. This first revealed that both metabolism and tissue regeneration were highly represented, conversely to what was found with the co-variance analysis, and that the overall pattern was similar to the pattern obtain from all DETs (Figure 1D). Comparisons with less sensitive networks, first layer of growth TFs group 2 PPI network and initial network of TFs group 4, are depicted in Supplementary Figure S8. The prediction of biological processes regulated by these networks failed to recapitulate the ones derived from DETs. This showed that a careful selection and control of the predicted networks should be performed.

We concluded that this third layer of growth TFs group 3 PPI network could be a good model of changes induced in cell networks after surgery and which led to the shift in expression of DETs. A deeper analysis of this network showed that the composition of the core network relied on STATs signalization pathways and was suggested to be highly dependent on Il-6 stimulation. Indeed, the induction of several major TFs regulating DETs such as STAT3, NFKB1, KLF4, SP1, SOCS3, STAT5a, STAT5b, REL, REL were linked to the Il-6 axis. In addition, Il-6 is known to stimulate immune cells recruitment to the injured tissue via the activation of signalization pathways like the PI3K and JAK/STAT pathways (Hartman et al., 2016; Tang et al., 2018). We thus assessed whether and when Il-6 was induced in the plasma of the same mice on which transcriptomic analysis was performed. As shown in Figure 5C, a significant and transient increase in blood Il-6 was detected 45 min post-surgery. These results suggest that Il-6 is very likely a major and early stimulus induced by the surgery stress at 45 min and which activates signalization pathways leading to the gene responses.

In conclusion, we propose an analytical pipeline for dynamic transcriptomic dataset which can be divided into two parts. First is the use of co-variance analysis of transcript expression leading to transcript clustering, combination of best transcripts modules prior to performing GO predictions. This method was efficient in discriminating modification of cell subtypes in the tissue like the one caused by immune cell infiltration or differentiation. Second, we used a retro-analysis strategy which starts with the prediction of the most potent TFs response elements from the all DETs and is followed by the simulation of TFs-based PPI network to predict the major biological processes upstream the TFs induction. We explained a way to test and select the most specific and sensitive predicted networks by comparing the GO terms predicted from simulated data (TFs) with the experimentally derived GO terms (from DETs).

By means of FACS analysis and ELISA assay measuring the level of circulating cytokines, we validated the main hypothesis raised from the predictions done from the transcriptomic dataset. Altogether, our results suggest that (i) Il-6 was induced by the surgery stress and likely initiated the tissue/cell responses and (ii) the surgery stress induced the recruitment of neutrophils and monocytes and the differentiation of hybrid M1/M2 macrophages as well. Since it was shown that Il-6 plays a major role in the neutrophils’ trafficking to the inflammation site (Kaplanski, 2003; Fielding et al., 2008), it is likely that Il-6 is activating the immune response in the sham hearts. Finally, this study demonstrates that the so-called “Sham” condition must be performed with a similar timing than the experimental conditions in order to be able to assess the surgery-based effects and discriminate it from the specific experimental effect. Indeed, both Il-6 involvement and inflammatory cells (neutrophils, monocytes/macrophages) recruitment have been reported in myocardial infarction, making the sham controls crucial to be performed for each and every time point. Once not considered, this could have led to an over-estimation of the effect of ischemia-reperfusion.

## Data Availability Statement

The datasets generated for this study can be found in Gene Expression Omnibus, GSE127244.

## Ethics Statement

This study was approved by the Ethics Committee of the Université Claude Bernard Lyon 1 (Approval Number DR2017-48) in compliance with NIH Guide on the Use of Laboratory Animals (NIH Publication No. 85-23, revised 1996).

## Author Contributions

SB, BP, JL, CC, and GB conceived the experiments. SB, AP, ZH, BP, LA, JL, CC, and GB performed the experiments. SB and GB wrote the manuscript. FV, MO, and GB supervised the study. JL, FV, MK, and MO reviewed the manuscript.

## Conflict of Interest

The authors declare that the research was conducted in the absence of any commercial or financial relationships that could be construed as a potential conflict of interest.

## Footnotes

1 https://string-db.org/

2 www.graphpad.com
