# Supplementary material for: A Dynamic Transcriptional Analysis Reveals IL-6 Axis as a Prominent Mediator of Surgical Acute Response in Non-ischemic Mouse Heart
Source: Front Physiol. 2019 Oct 31;10:1370. doi: 10.3389/fphys.2019.01370 (PMC6836931; doi:10.3389/fphys.2019.01370)
Supplement: Supplementary file 1 [file Data_Sheet_1.pdf]

Supplementary Material

1. Supplementary Tables and Figures

Supplementary Table S1: List of antibodies used in flow cytometry analysis.

Antibodies	Supplier	References	Isotypic control	Supplier	References
PE-F4/80	Miltenyi biotec	130-102-422	REA control-PE	Miltenyi biotec	130-104-628
VIOBLUE - CD11B	Miltenyi biotec	130-097-336	Rat IgG2b-Vioblue	Miltenyi biotec	130-102-661
APC-CD86-	Miltenyi biotec	130-102-558	Rat IgG2b-APC	Miltenyi biotec	130-102-664
AF488-CD206	Biolegend	BLE141710	AF488 Rat IgG2a	Biolegend	BLE400525
Anti Ly6g-FiTC	Miltenyi biotec	130-107-913	Rat control-PE	Miltenyi biotec	130-104-628

Supplementary Table S2: List of differentially expressed transcripts by DESeq2 analysis.

Supplementary Table S3: List of transcripts assigned to different WGCNA modules.

Supplementary Table S4: List of enriched GO terms for DETs, WGCNA studied modules.

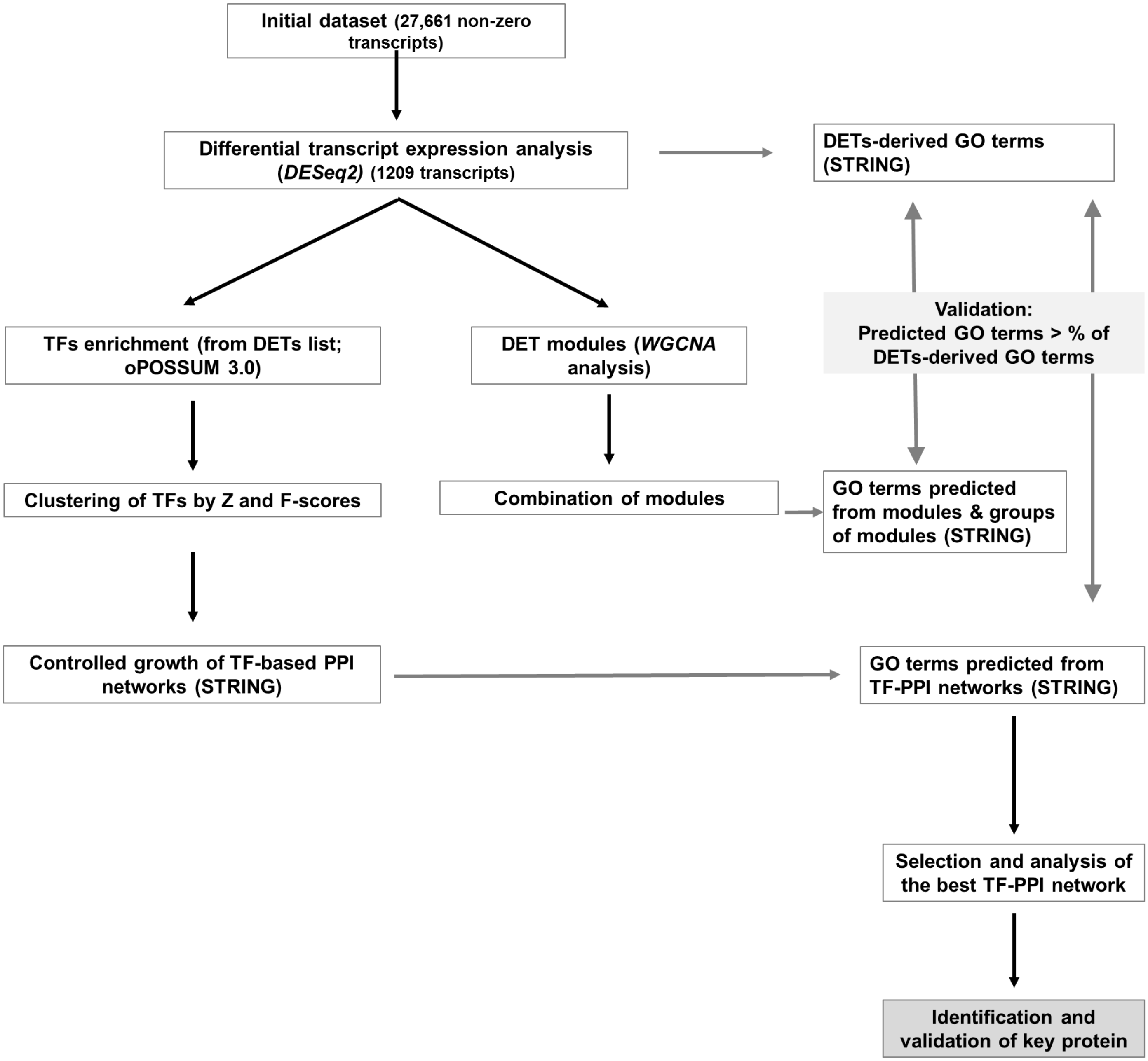

Supplementary Figure 1: Data analysis workflow

Sequencing data (27,661 non-zero transcripts) were analyzed by *DESeq2* R package for differential transcript expression (DET) detection. DETs were then either clustered in gene modules by *WGCNA* R package based on their variation over time or submitted to oPOSSUM to predict the TFs which regulate the DETs expression. The predicted TFs were used as input for STRING simulation aiming to reconstruct the protein-protein interaction (PPI) networks upstream of the TFs. Both transcript

modules and PPI-networks were analyzed functionally (GO analysis). Analysis data were integrated and targets of interest were identified.

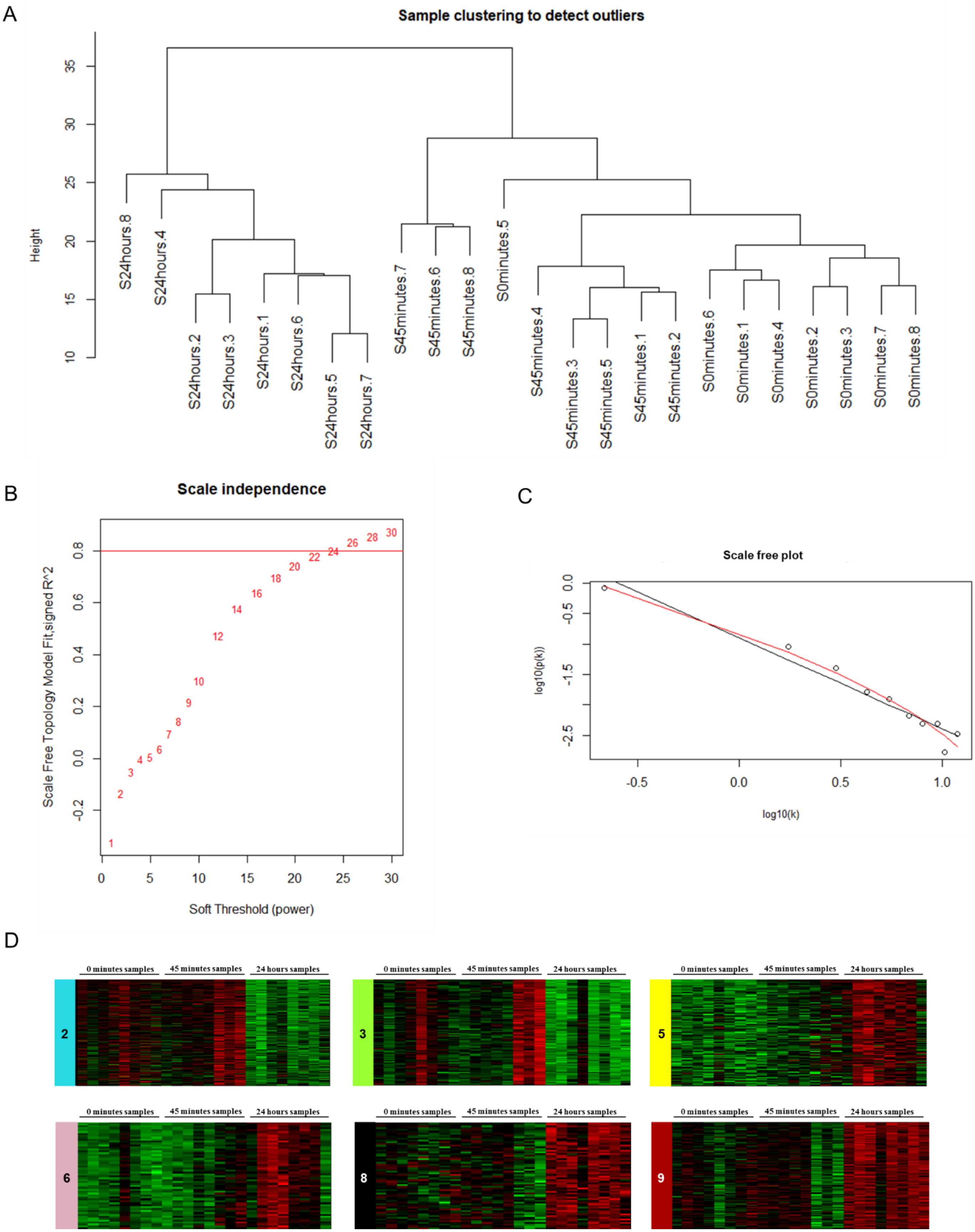
A
Sample clustering to detect outliers
Height
10
15
20
25
30
35
S24hours.8
S24hours.4
S24hours.2
S24hours.3
S24hours.1
S24hours.6
S24hours.5
S24hours.7
S45minutes.7
S45minutes.6
S45minutes.8
S0minutes.5
S45minutes.4
S45minutes.3
S45minutes.5
S45minutes.1
S45minutes.2
S0minutes.6
S0minutes.1
S0minutes.4
S0minutes.2
S0minutes.3
S0minutes.7
S0minutes.8
B
Scale independence
Scale Free Topology Model Fit,signed R^2
Soft Threshold (power)
C
Scale free plot
log10(p(k))
log10(k)
D
0 minutes samples
45 minutes samples
24 hours samples
2
3
5
6
8
9

Supplementary Figure 2: WGCNA quality checks

(A) Samples' clustering tree by average method to identify outlier samples. No outlier samples were detected. (B) Soft threshold (power) determination based on topology fitting results. Horizontal red line corresponds to model fil of R^2 0.9. (C) A scale free topology log-log plot showing the whole network connectivity distribution. The logarithm of the network connectivity and the corresponding frequency distribution are shown in the x-axis and y-axis, respectively. Black line corresponds to the linear regression line of R^2: 0.94. An enhanced R^2 (0.96) is obtained by the fit of truncated power law shown by the red line based on the chosen power (power=30). (D) Heatmaps for the genes assigned to the modules: 2, 3, 5, 6, 8 and 9 in the 24 samples (red: higher expression, green: lower expression).

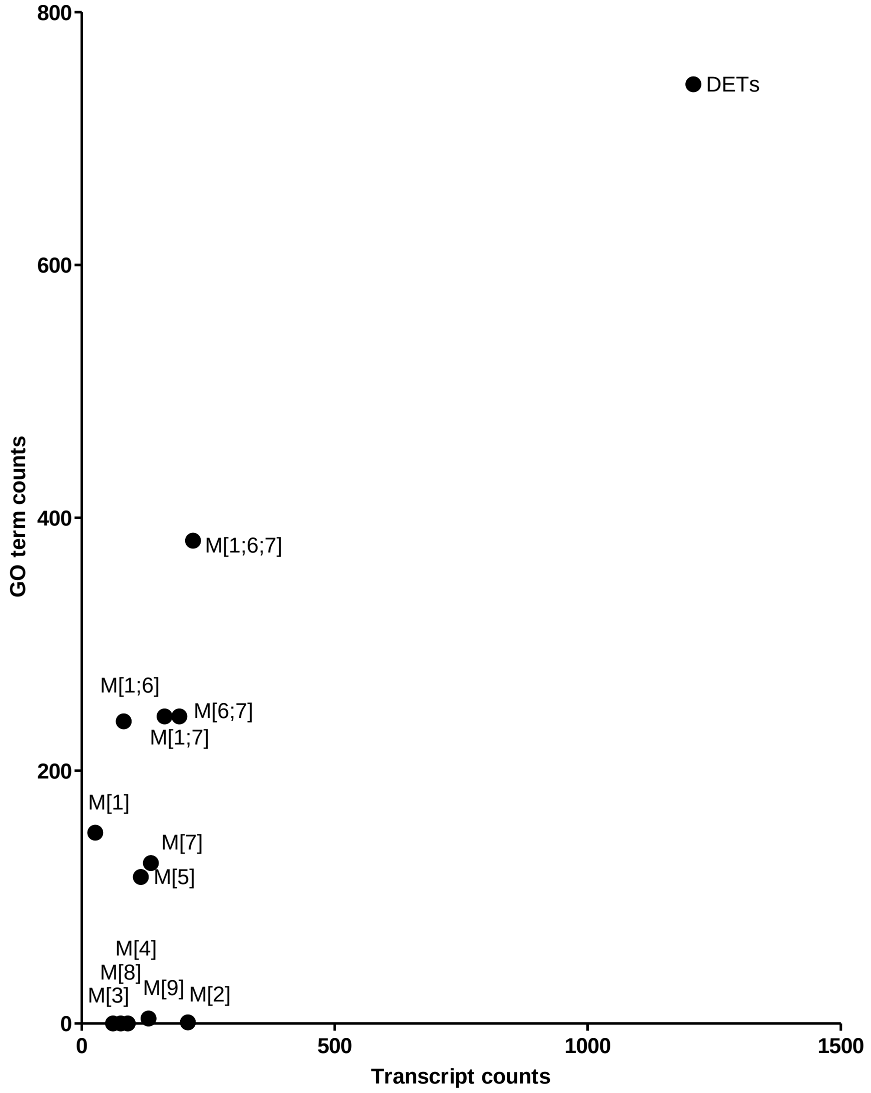

Supplementary Figure 3: **GO terms and WGCNA data integration**

Dot plot represents the counts of GO terms predicted for each transcripts modules or all DETs as a function of the count of transcripts.

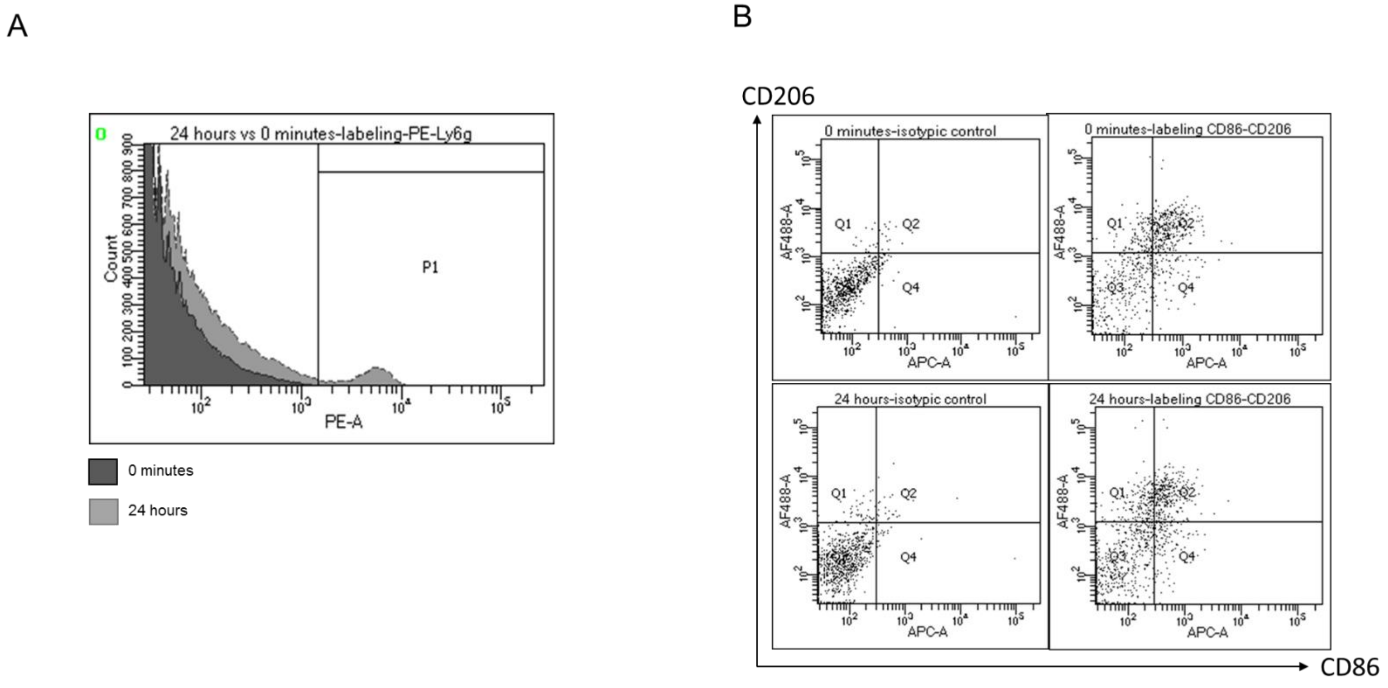

Supplementary Figure 4: Flow cytometry

(A) Distribution plot of LY6-G positive cells in 0 min compared to 24 hours samples analyses by Flow cytometry. LY6-G positives cells are gated in P1. (B) Scattering plots of double-labeled cells analysis comparing 0 min vs 24h samples and corresponding isotypic control. Cells are gated for analysis according to their respective fluorescent channels (APC for CD 86 and AF488 for CD 206) in 4 distinct gates: Q1 corresponding to CD 206 positive/ CD 86 negative cells, Q2 double positive cells, Q3 double negative cells and Q4 CD 86 positive/CD 206 negative cells.

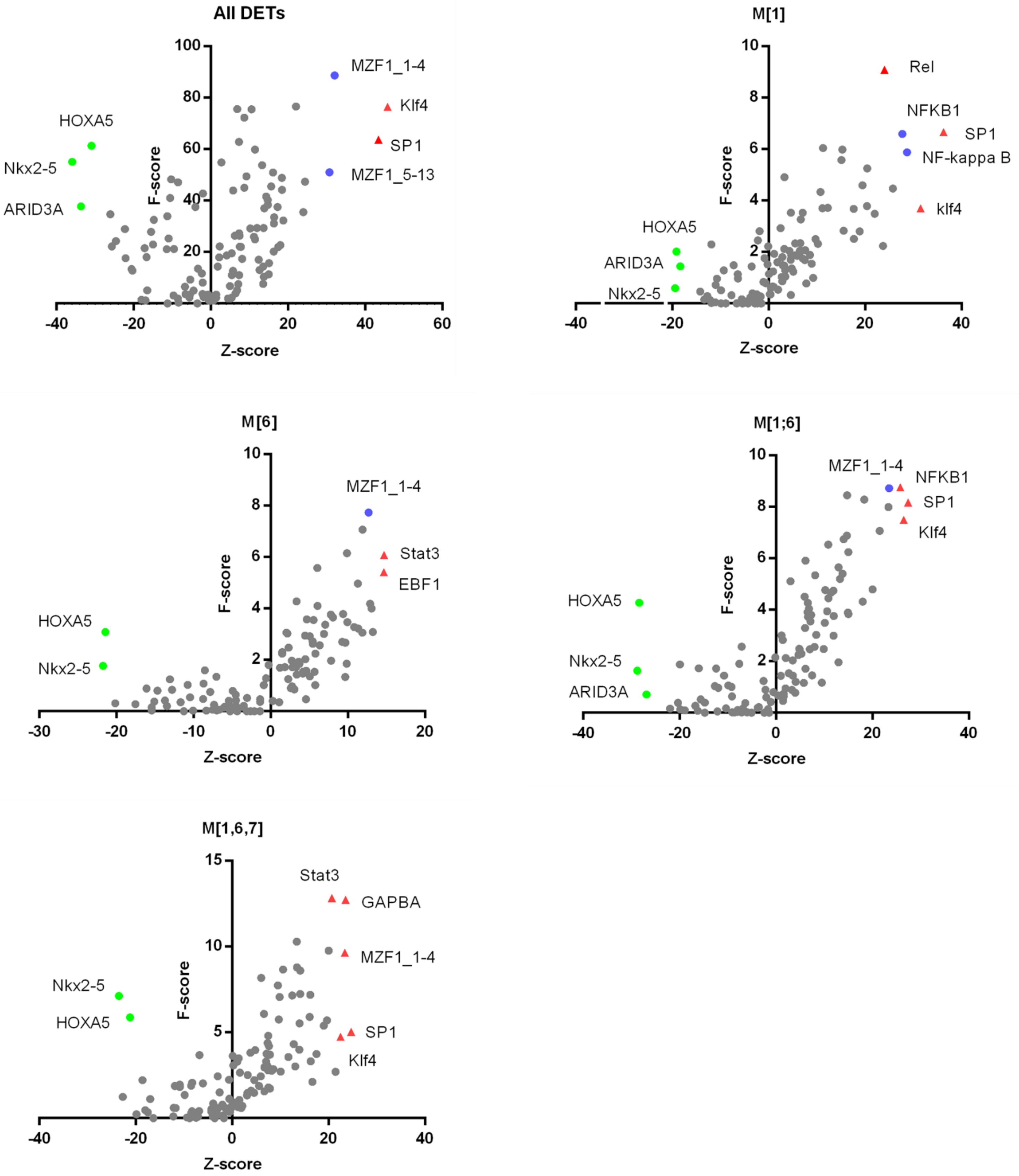
All DETs
MZF1_1-4
Klf4
HOXA5
SP1
Nkx2-5
MZF1_5-13
ARID3A
F-score
Z-score
M[1]
Rel
NFKB1
SP1
NF-kappa B
klf4
HOXA5
ARID3A
Nkx2-5
F-score
Z-score
M[6]
MZF1_1-4
Stat3
EBF1
HOXA5
Nkx2-5
F-score
Z-score
M[1;6]
MZF1_1-4
NFKB1
SP1
Klf4
HOXA5
Nkx2-5
ARID3A
F-score
Z-score
M[1,6,7]
Stat3
GAPBA
MZF1_1-4
Nkx2-5
HOXA5
SP1
Klf4
F-score
Z-score

Supplementary Figure 5: Transcription factor enrichment analysis of the WGCNA assigned modules.

Represents scatter plots of Z-score and F-score of transcription factor binding site (TFBS) of all DETs, genes assigned to the magenta module (M[1]), pink module (M[6]), group of magenta and pink modules (M[1;6]), group of pink and blue modules (M[6;7]) and group of magenta, pink and blue modules (M[1;6;7]). Over-represented TFs were shown in color in order to compare their detection in the different modules and in all DETs.

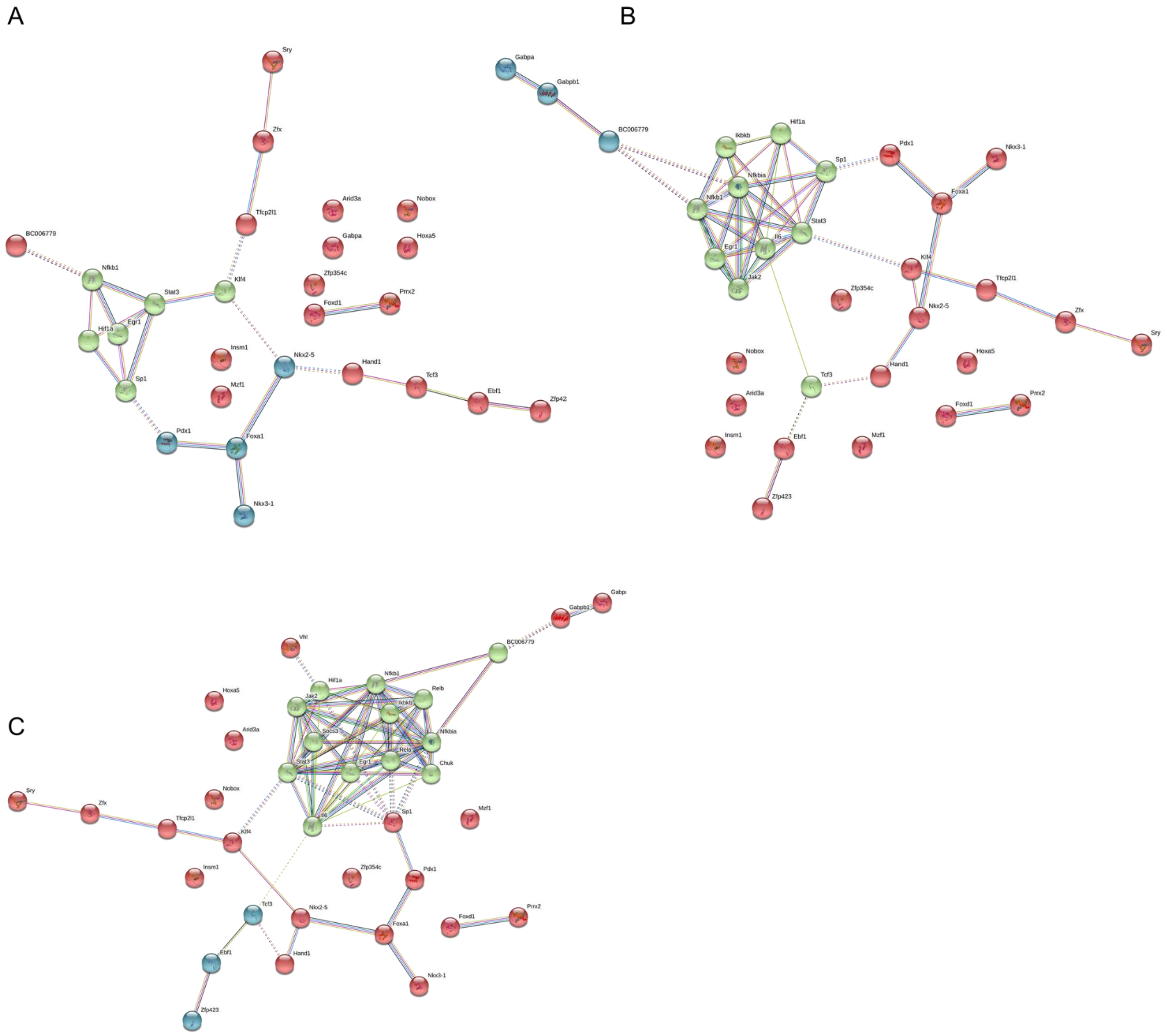

Supplementary Figure 6: TFs-regulated network expansion in STRING

Schemes display the growth of the STRING initial TF-PPI network (A) of TFs group 3 by two consecutive addition of protein neighbors (+5 at each layer) as shown in (B) and (C), respectively.

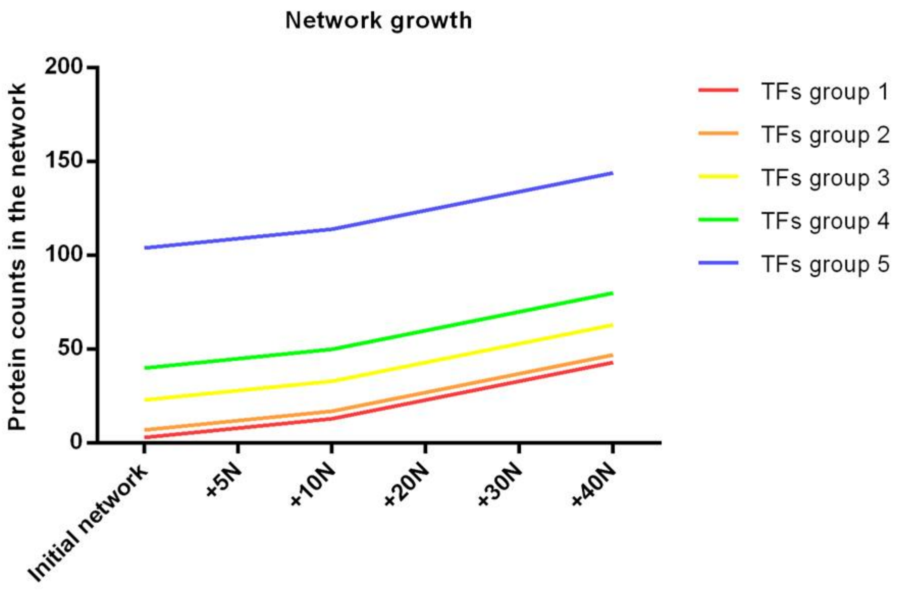

Supplementary Figure 7: TF enrichment analysis

Line graph displaying the increase in the count of proteins in each group of TF-PPI networks after the addition of protein neighbors in five consecutive steps. X axis displays the cumulated count of added neighbors from the initial network. TF-PPI networks were generated using the 5 different groups of TFs as input.

A

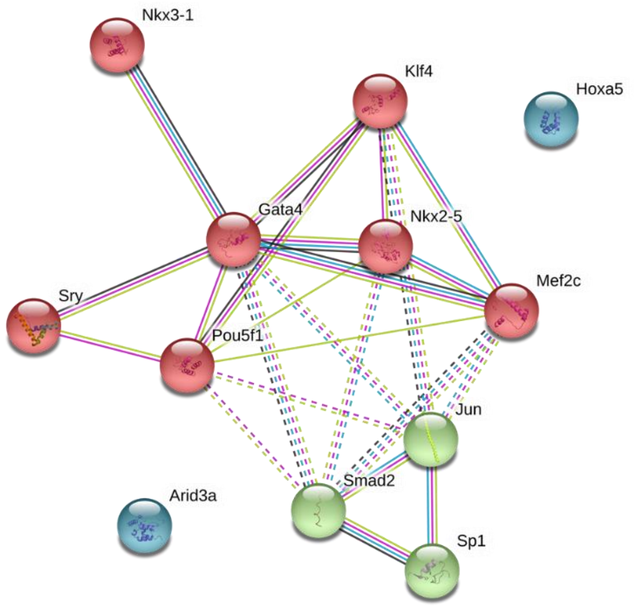

1st neighborhood growth of TF group 2 network

C

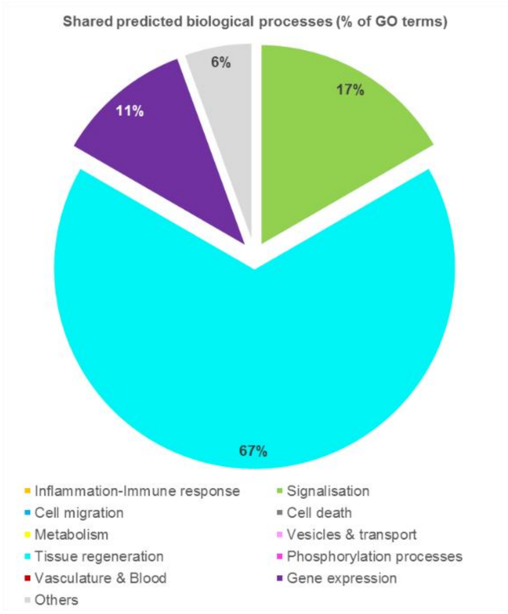

B

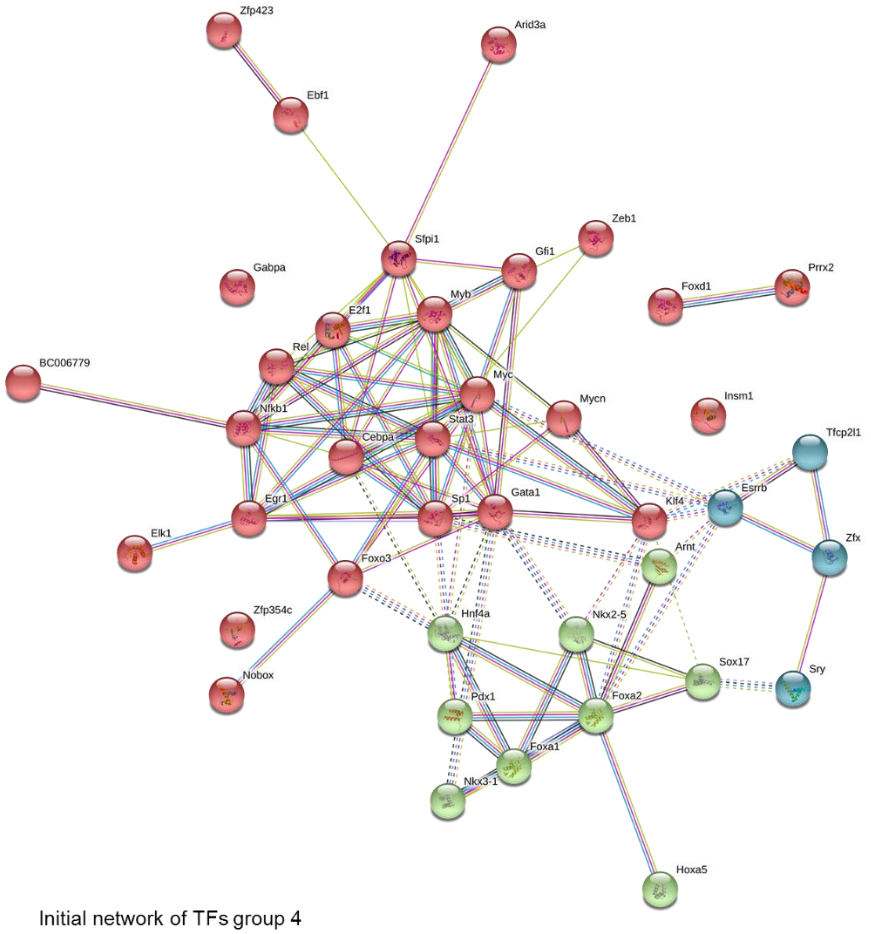

Initial network of TFs group 4

D

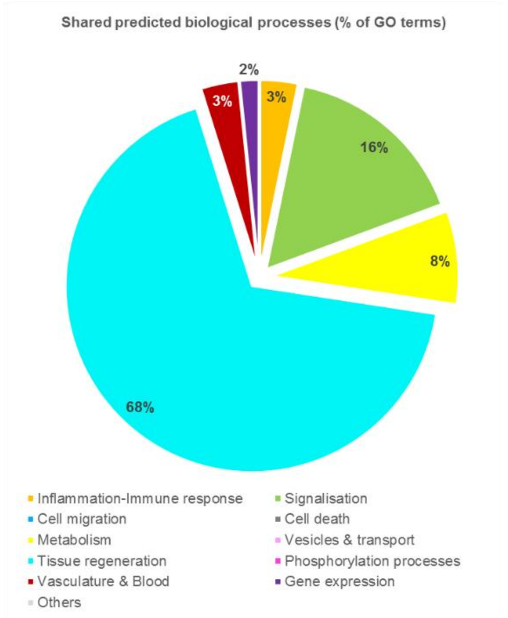

Supplementary Figure 8: TFs-regulated networks and their predicted GO terms

(A) and (B) show graphical representations of TF-PPI networks of TFs group 2 expanded for +10 neighbors and TFs group 4 initial network, respectively. (C) and (D) Pie charts showing the distribution of GO terms derived from the proteins of the networks shown in (A) and (B), respectively, and classified in bigger biological processes.
